# Biphasic clinical course of a ruptured right gastric artery aneurysm caused by segmental arterial mediolysis: a case report

**DOI:** 10.1186/s12893-020-00849-x

**Published:** 2020-08-27

**Authors:** Yuichiro Kohara, Koji Fujimoto, Hikotaro Katsura, Takashi Komatsubara, Kazuhito Ichikawa, Hiroshi Higashiyama

**Affiliations:** 1 Department of gastrointestinal surgery, Shinko Hospital, 1-4-47 Wakinohama-cho, Chuo-ku, Kobe, 651-0072 Japan; 2 Department of pathology, Shinko Hospital, 1-4-47 Wakinohama-cho, Chuo-ku, Kobe, 651-0072 Japan

**Keywords:** Segmental arterial mediolysis, Case report, Aneurysm, Right gastric artery, Double-rupture phenomenon

## Abstract

**Background:**

Gastric artery aneurysms are rarely caused by segmental arterial mediolysis (SAM), a condition that often involves multiple vessels. The clinical course of SAM after vessel rupture may vary depending on the involved vessels. For example, the “double-rupture phenomenon” observed following the rupture of the splenic artery aneurysm manifests as a biphasic and relatively slow clinical course. Even in cases of rupture of gastric artery aneurysm, the double-rupture phenomenon has only been reported in two cases so far. However, the rupture was not caused by SAM in either case. Herein, we present the apparent first case of a right gastric artery (RGA) aneurysm rupture caused by SAM that presented with a biphasic clinical course, possibly due to the double-rupture phenomenon.

**Case presentation:**

A 54-year-old woman was transferred to the emergency department with severe abdominal pain and a cold sweat for a duration of 3 h. She had developed mild abdominal pain and nausea 3 days earlier. Her vital signs were stable. Physical examination revealed tenderness in the epigastric area. Abdominal contrast-enhanced computed tomography revealed an RGA aneurysm with contrast media extravasation. A diagnosis of hemoperitoneum following a ruptured RGA aneurysm was made, and the patient underwent angiography. However, this modality did not reveal any extravasation from the RGA due to an interruption in the peripheral branch of the artery. Nevertheless, to prevent major bleeding, we performed coil embolization at the point of interruption in the RGA, which we suspected to be a ruptured aneurysm. A distal gastrectomy with Roux-en-Y reconstruction for aneurysm resection was performed the following day. There were no postoperative complications, and the patient was discharged 17 days after surgery. Histologically, the RGA demonstrated multiple vacuoles in the medial muscle layer, which were characteristic of SAM.

**Conclusions:**

An RGA aneurysm rupture should be considered a differential diagnosis in patients presenting with hemoperitoneum with a slow or biphasic clinical course.

## Background

Gastric artery aneurysm caused by segmental arterial mediolysis (SAM) is extremely rare. Rarer still, is the rupture of this type of aneurysm, with only one other reported case to date [1]. SAM is a nonatherosclerotic, and noninflammatory disease of the arterial media that often involves multiple vessels [2]. In such cases, the clinical course following a rupture may vary depending on the involved vessels [3]. For example, the rupture of a splenic artery aneurysm exhibits a biphasic and relatively slow clinical course described as the “double-rupture phenomenon” [4]. This phenomenon is attributable to the initial rupture of the splenic artery aneurysm into the omental bursa, which forms a hematoma that has a tamponade effect on bleeding. A second rupture into the peritoneal cavity might be attributable to a variation in intra-abdominal pressure 6–96 h later [4, 5]. Previous reports have estimated an incidence of the double-rupture phenomenon ranging from 12 to 21% among patients with rupture of the splenic artery aneurysm [4, 6].

Even in cases of rupture of gastric artery aneurysm, the double-rupture phenomenon was only reported in two cases so far [7, 8]. However, the rupture was not caused by SAM in either case. Herein, we report a case of right gastric artery (RGA) aneurysm rupture caused by SAM that presented with a biphasic clinical course, possibly due to the double-rupture phenomenon.

## Case presentation

**Fig. 1:**
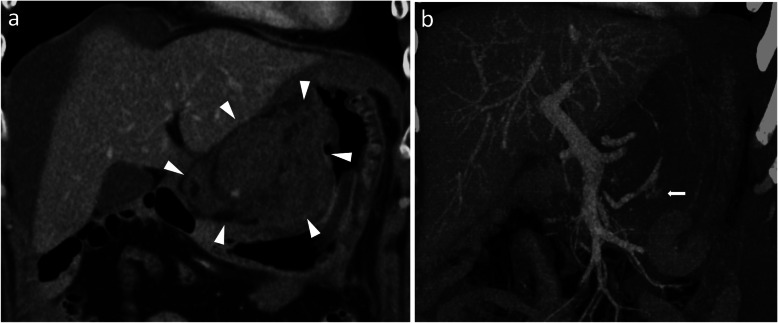
**a**: Abdominal computed tomography showed a mass on the lesser curvature (white triangles) that showed enhancement in the early phase. **b**: There was extravasation of the contrast media from the right gastric artery in the late phase (white arrow)

**Fig. 2:**
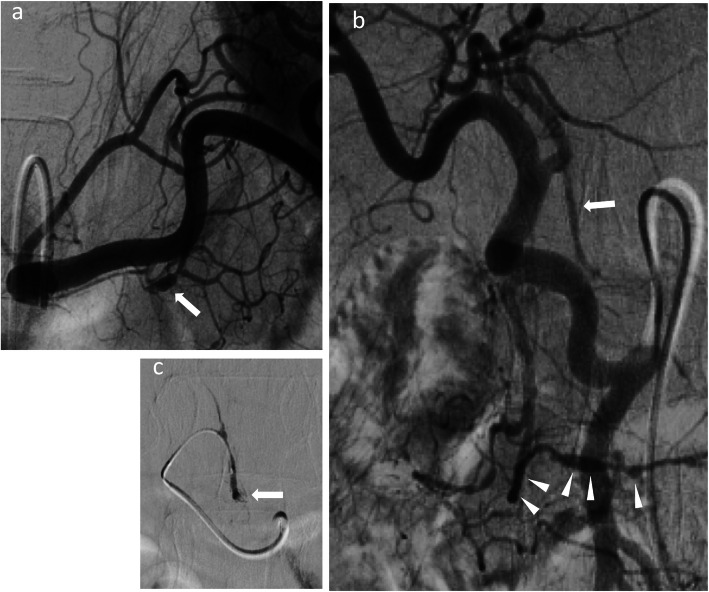
**a**: Angiography revealed aneurysmal dilatation of the branch of the left gastric artery (white arrow). **b**: The replaced common hepatic artery originated from the superior mesenteric artery. The right gastric artery (RGA) arose from the left hepatic artery (white arrow). The right gastroepiploic artery had the appearance of ‘string of beads sign’, which was characteristic of segmental arterial mediolysis. (white triangles). **c**: There was interruption in the peripheral branch of the RGA (white arrow)

**Fig. 3:**
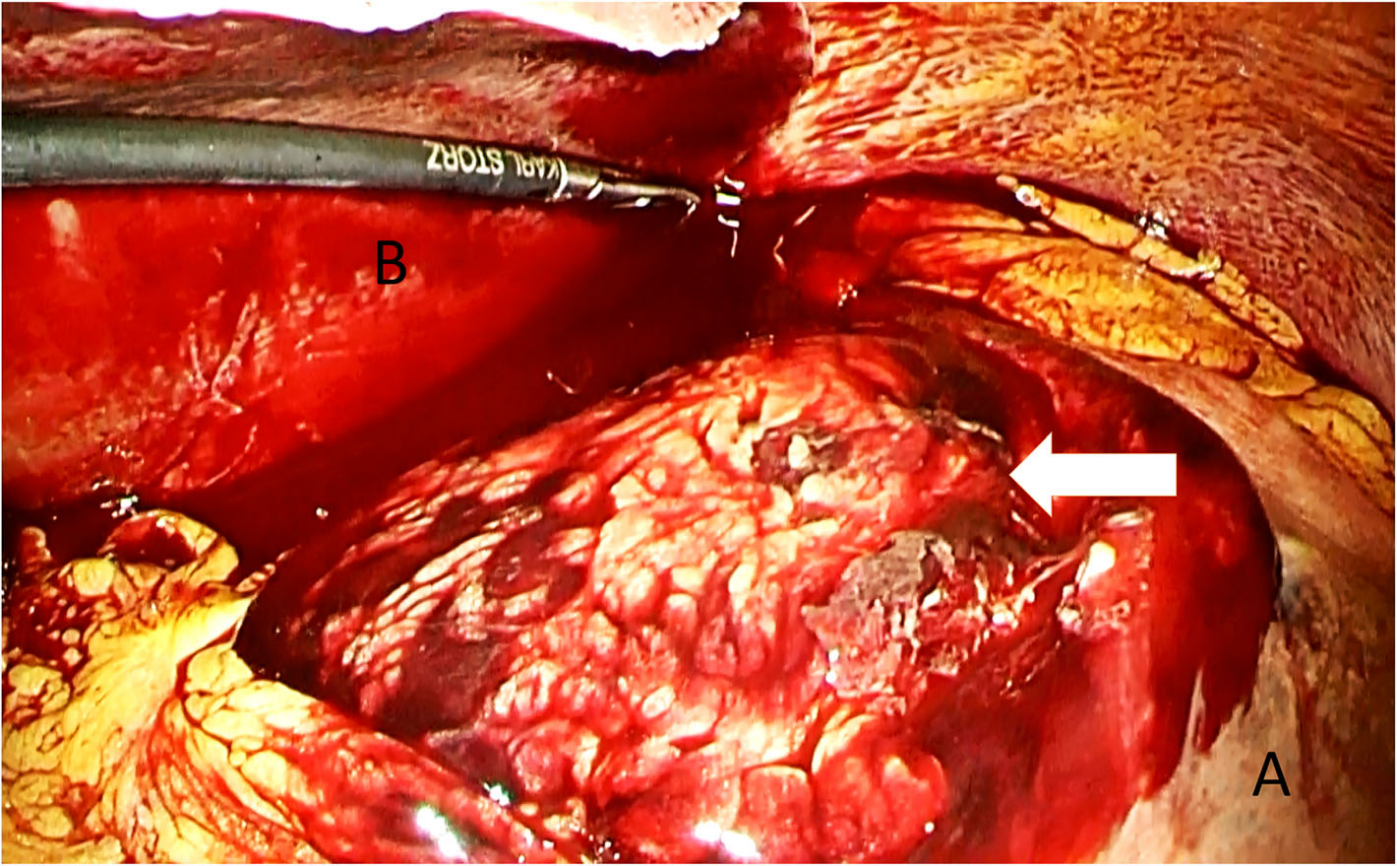
There was the massive hematoma in the lesser omentum (white arrow). *a: Liver, **b**: Stomach

A 54-year-old woman was transferred to the emergency department with complaints of severe abdominal pain and a cold sweat that had developed 3 h earlier. She had developed mild abdominal pain and nausea 3 days earlier. She had no other relevant medical history. Her vital signs were as follows: blood pressure, 116/84 mmHg; pulse rate, 74 beats/min; and body temperature, 35.7 °C. Physical examination revealed tenderness in the epigastric area. Her laboratory tests were normal except for a low hemoglobin level (10.9 mg/dL). Abdominal contrast-enhanced computed tomography (CT) showed a mass on the lesser omentum with enhancement in the early phase, and the liver surface was surrounded by high-density ascites (Fig. 1a). Extravasation of the contrast media from the RGA (Fig. 1b) was also visible. A diagnosis of hemoperitoneum consequent to the rupture of an RGA aneurysm was made. She subsequently underwent angiography, which revealed aneurysmal dilatation of the branch of the left gastric artery (LGA) and right gastroepiploic artery (RGEA) (Fig. 2a, b). The interruption in the peripheral branch of the RGA precluded the detection of extravasation from this artery via angiography (Fig. 2c). Because we suspected a rupture of the RGA aneurysm, we performed coil embolization at the point of interruption in the RGA to prevent major bleeding. However, her hemoglobin levels continued to decline on the following day. Considering her continuous bleeding, we performed a diagnostic laparoscopy which revealed a lot of bloody ascites in the peritoneal cavity. Additionally, a massive hematoma was found in the lesser omentum (Fig. 3). We converted to laparotomy due to insufficient view because of the massive hematoma, and performed a distal gastrectomy with Roux-en-Y reconstruction to resect the aneurysms in the RGA, LGA, and RGEA. There were no postoperative complications and the patient was discharged 17 days after surgery.

**Fig. 4:**
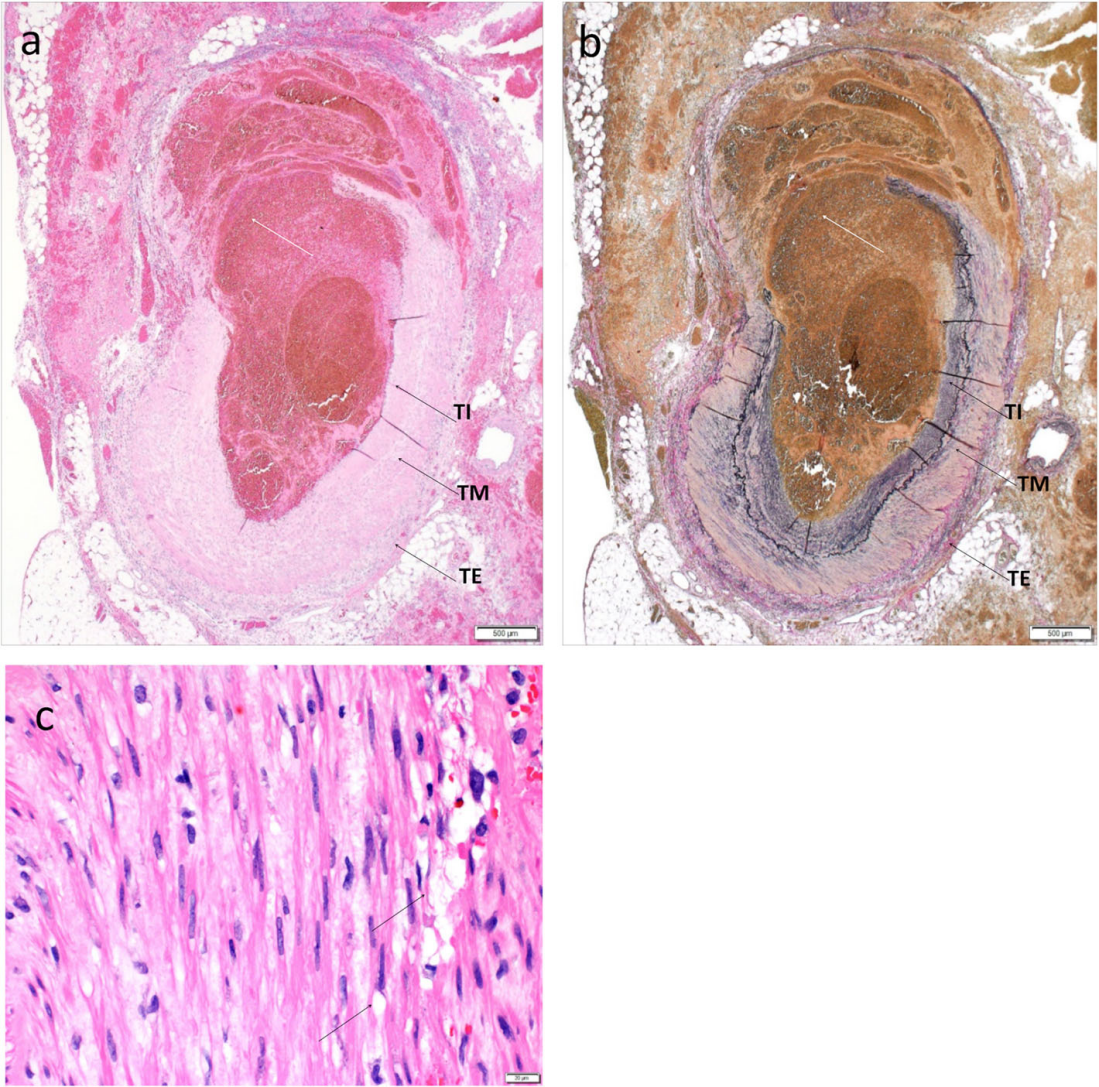
**a** (H. E. staining) and **b** (E.V.G staining): Tunica media and intima were partially disrupted (white arrow). **c**: Multiple vacuoles were displayed in the medial muscle layer. *TE (tunica externa), TM (tunica media), TI (tunica intima)

A pathological examination of the resected RGA revealed partial dissection of the tunica media and intima and a hematoma that had filled the dissected cavity, indicating rupture of the RGA aneurysm (Fig. 4a, b). Multiple vacuoles, a characteristic of SAM, were seen in the medial muscle layer (Fig. 4c). Similar findings were observed in the resected specimens of the LGA and RGEA. Based on these findings, we made a final diagnosis of a ruptured RGA aneurysm caused by SAM.

## Discussion and conclusion

In this report, we described the case of a 54-year-old woman who was diagnosed with a ruptured RGA aneurysm and presented with a biphasic clinical course possibly indicative of the double-rupture phenomenon, which is typically observed with splenic artery aneurysm.

More recently, advances in diagnostic modalities, including CT and histology, have led to an increased number of reports of visceral artery aneurysms caused by SAM. Based on a study of 117 cases of SAM, Peng et al. reported an average patient age of 57.2 years and a male predominance (67.5%) among affected patients. The most commonly involved vessel was the celiac artery, including the celiac axis and its branches. Overall, 4.3% of patients in that study died, although only one death was SAM-related [3]. However, the reported mortality varies widely, as other studies have described mortality rates as high as 50% in patients with acute hypotension secondary to SAM [9]. This large disparity in mortality rates has been attributed to recent improvements in diagnostic and therapeutic techniques in a current review [10], as well as the inherent reporting bias wherein only cases involving the most severe forms of rare diseases, such as SAM, tend to be reported [3]. Therefore, few reports have described cases of visceral aneurysm rupture that presented with a relatively slow clinical course, as in our case. When searching on PubMed for relevant literature, we found only one detailed report of a case of gastric artery aneurysm rupture caused by SAM. However, that patient was already critically ill with hypotension upon arrival at the hospital and received a diagnosis of LGA rupture. No reports have described the rupture of an RGA aneurysm caused by SAM.

Despite the rupture of a visceral artery aneurysm, our patient exhibited a biphasic and relatively slow clinical course comparable to a case of splenic artery aneurysm rupture with the double-rupture phenomenon reported by Bokerman in 1930 [4]. Two other clinical reports described the double-rupture phenomenon during the rupture of an LGA aneurysm [7, 8]. The clinical course of our case was similar to the courses of these previous cases. During the rupture of a gastric artery aneurysm, the initial bleeding might spread to the lesser omentum, followed by a second rupture caused by a tear in the serous membrane into the omental bursa or peritoneal cavity [7]. In our case, the initial bleeding into the lesser omentum might have caused the mild abdominal pain experienced by the patient 3 days before presentation, while the second rupture into the peritoneal cavity resulted in the sudden onset of severe abdominal pain 3 h prior to presentation.

It was difficult to confirm the double-rupture phenomenon in our present case, as imaging studies did not reveal the RGA aneurysm rupture prior to the second bleeding, which spread into the peritoneal cavity. The lack of evidence of a rupture on imaging studies is a limitation of this report. We arrived at a diagnosis of ruptured RGA aneurysm with a double-rupture phenomenon based on the clinical course and the hematoma in the lesser omentum observed on imaging. Few reports have explored this phenomenon other than earlier literature on the ruptures of splenic artery aneurysms. Therefore, the detailed mechanism of the onset of the double–rupture phenomenon remains poorly understood. In this situation, timely diagnosis and management of the ruptured RGA aneurysm prior to hemodynamic instability caused by the second bleeding is crucially important. This case can serve as a reference for the diagnosis of a ruptured RGA aneurysm and the planning of an appropriate treatment strategy, which may include laparotomy, laparoscopic surgery, transcatheter arterial embolization, and other modalities. However, further case reports are needed to clarify the clinical features of a ruptured RGA aneurysm.

We reported herein a case of a ruptured RGA aneurysm caused by SAM that exhibited a biphasic clinical course, possibly caused by the double-rupture phenomenon. An RGA aneurysm rupture must be included in the differential diagnosis of a patient with hemoperitoneum who presents with a slow or biphasic clinical course.

## Data Availability

The datasets supporting the conclusions of this article are included within the article.

## Abbreviations

SAM: Segmental arterial mediolysis
RGA: Right gastric artery
CT: Computed tomography
LGA: Left gastric artery
RGEA: Right gastroepiploic artery
